# Estrogen-mediated corneal collagen degradation in keratoconus

**DOI:** 10.1016/j.isci.2025.113004

**Published:** 2025-06-25

**Authors:** Amit Chatterjee, Levi N. Kanu, Nikolay Boychev, Amy E. Ross, Vincent Yeung, Nandini Venkateswaran, Hajirah N. Saeed, Joseph.B. Ciolino

**Affiliations:** 1 Department of Ophthalmology, Schepens Eye Research Institute of Mass Eye and Ear, Harvard Medical School, Boston, MA 02114, USA; 2 Department of Ophthalmology and Visual Sciences, University of Illinois, Chicago, IL, USA

**Keywords:** Biochemistry, Cell biology

## Abstract

Keratoconus (KC) is characterized by corneal thinning due to pathologic degradation of corneal stromal collagen. This study investigates the role of β-estradiol in corneal collagen degradation using both tear fluid samples from KC and non-KC patients and *in vitro* models (co-culture and 3D collagen hydrogel model). In tear fluids, we observed sex-specific differences in matrix metalloproteinase (MMP) activity, secretory phospholipase A2 (PLA2G2A) levels, and in cytokine profiles, including interleukin (IL-) 2, 3, 5, 6, 8, 11, 13, 15, 19, 24, 27, 31, and 32 tumor necrosis factor (TNF-α), angiopoietin 2, vitamin D binding protein, insulin growth factor binding proteins (IGFBP3), vascular endothelial growth factor (VEGF), and receptor for advanced glycation end product (RAGE). Our model showed that both corneal epithelium and fibroblasts synthesize their own estrogen, and β-estradiol treatment via p38 MAP kinase pathway regulates MMP2-mediated collagen fiber degradation. p38 MAP kinase inhibitor SB202190 significantly reduced β-estradiol-induced MMP activity and collagen breakdown, as well as cytokine regulation suggesting a potential therapeutic approach.

## Highlights

• Estrogen, sPLA2, and NGAL-MMP9 levels are elevated in tears from female KC patients
• Estrogen triggers collagen degradation via MMP2 activation through the p38 MAP kinase pathway
• 3D hydrogel model of cornea for quantifying estrogen-mediated collagen fiber degradation
• SB202190 mitigates estrogen-mediated collagen degradation

## Introduction

Keratoconus (KC) is a progressive corneal ectasia characterized by corneal thinning and protrusion, leading to visual impairment.^1^^,^^2^ While the pathogenesis of KC is multifactorial, involving a complex interplay of biochemical, immunological, metabolic, and genetic factors,^3^^,^^4^^,^^5^^,^^6^^,^^7^ there is increasing evidence that hormones may play a role in progression of the disease. Elevation in systemic hormone levels (e.g., androgen,^8^ estradiol,^7^^,^^9^ luteinizing hormone/follicle-stimulating hormone levels,^10^ prolactin, and thyroid hormones^8^^,^^11^^,^^12^) have been observed in patients with KC. Moreover, KC often first manifests during or after puberty—when sex hormone levels change significantly—and KC has been shown to progress in patients undergoing hormone replacement therapy who are older and therefore would otherwise be at a low risk for KC progression.^13^ Clinical studies have revealed that pregnancy, which is also associated with changes in hormone levels, induces significant differences in corneal biomechanics and topographic parameters.^14^ Research in organ culture models has shown that the cornea is a hormone-responsive tissue and estrogen treatment is independently sufficient to affect the Young’s modulus and the thickness of the porcine cornea.^15^ However, the mechanisms by which extragonadal hormones contribute to the development of KC remain largely unexplored.

Pathological mechanisms in KC involve stromal collagen degradation, keratocyte apoptosis, inflammation, and activation of several enzymes involved in the remodeling of extracellular matrix (ECM). Matrix metalloproteases (MMP) has been extensively studied in KC corneas and tear fluid.^16^ KC patients’ tears have been reported to have elevated MMP9 levels.^17^^,^^18^ Also, KC stromal cells have been reported to have significantly upregulated MMP2 activity which causes stromal collagen breakdown.^19^ In addition to directly remodeling the ECM, MMP2/9 influence ECM metabolism through inflammatory mediators such as PLA2G2A, which regulates the expression of proinflammatory cytokines such as transforming growth factor β (TGF-β), interleukin-6 (IL-6), and tumor necrosis factor alpha (TNF-α).^18^ Previous studies on retinal pigment epithelium^20^ and human corneal epithelial cells^21^ have shown that β-estradiol treatment causes increases in MMP2, MMP9, and various other inflammatory genes but the mechanism by which it regulates MMP activity and cause collagen breakdown is not well understood as these genes do not have estrogen responsive elements in their promoter region.^22^ Previous phospho-proteomic studies have showed that in KC epithelium mitogen-activated protein kinase pathways (p38 MAP kinase, p44 MAP kinase) were upregulated.^23^ However, whether sex steroid hormones (e.g., estrogen) regulate the p38 or p44 MAP kinase pathways—and thereby influence MMP activity and contribute to stromal collagen breakdown—remains to be fully elucidated. Elucidating the mechanism by which sex hormones regulate the spatiotemporal MMP9/2 activity and cause collagen breakdown warrants further investigation.

Although various signaling pathways relevant to KC have been identified using multi-omics approaches (Wnt signaling pathway, focal adhesion pathway, ECM remodeling pathway, and interleukin-6-mediated inflammatory pathway^24^^,^^25^), the lack of a universally recognized animal model that recapitulates the disease pathology hinders the evaluation of causal factors and potential therapeutics.^26^ Thus, in the absence of an accepted animal model, 3D cell culture models using the required cell types and the extracellular matrix component may serve as an excellent tool to study the disease pathogenesis.^27^ Published *in vitro* models used for studying KC pathophysiology include keratocytes or fibroblasts isolated from cadaveric and KC corneas and growing them on ECM proteins,^28^ or treating them exogenously with TGF-β^28^^,^^29^ or epithelium collected from patients during collagen crosslinking.^17^^,^^23^^,^^24^ However, none of these models have been able to pinpoint the cell-specific causal role or the spatiotemporal regulations involved in the breakdown of collagen fibers. Thus, we developed a 3D corneal hydrogel model (for quantifying collagen fiber degradation) and co-culture model that both use corneal epithelium, primary corneal fibroblast and collagen to identify the cell specific causal role in the disease progression in response to exogenous β-estradiol treatment, a contributing factor in KC pathology.

The aim of the present study is to investigate possible mechanisms linking sex hormones and collagen degradation in KC. We initially describe sex-specific differences in estrogen levels, MMP activity, PLA2G2A levels, and cytokine levels in the tear fluids of KC patients and healthy controls. We further explore the mechanism by which β-estradiol modulates the PLA2G2A-MMP2/9-MAP kinase pathway, leading to collagen breakdown, utilizing co-culture and 3D collagen hydrogel models. This research seeks to enhance our understanding of the hormonal influences on KC pathology and the potential for targeted therapeutic strategies.

## Results

### Elevated estrogen, secretory phospholipase A2, and MMP activity levels in tears from KC females

**Figure 1:**
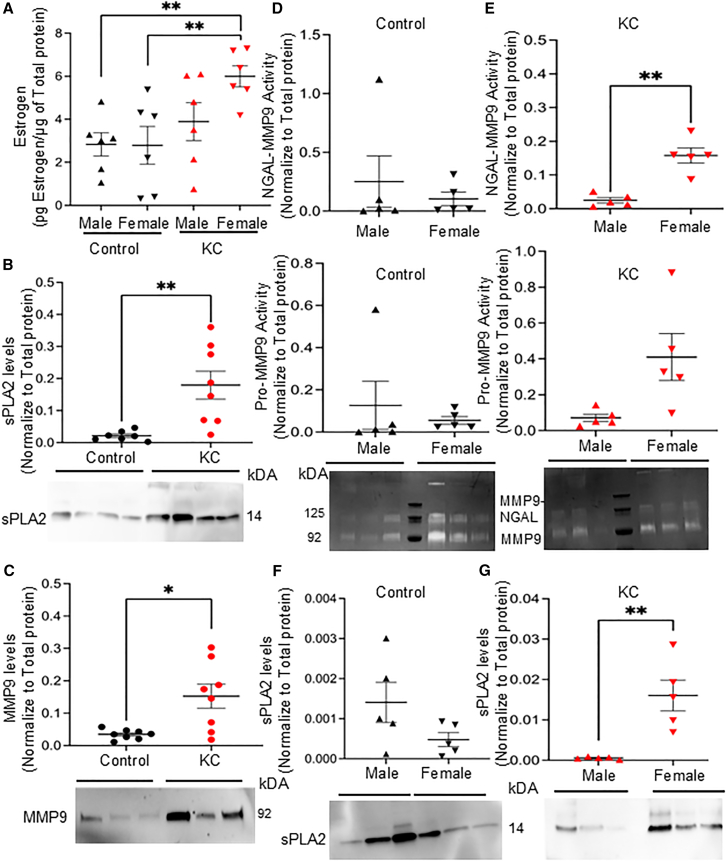
Elevated estrogen, secretory phospholipase A2 and MMP activity levels in tears from KC females (A) Estrogen ELISA of tear fluids collected from KC and non- KC (male vs. female patients) (*N* = 6/group), ∗*p* < 0.05; ∗∗*p* < 0.01 one-way ANOVA. (B) Immunoblotting of PLA2G2A of individual tear fluids collected from KC and non- KC patients (*N* = 8/group), ∗*p* < 0.05; ∗∗*p* < 0.01 Student’s t test. (C) Immunoblotting of MMP9 of tear fluids collected from KC and non- KC patients (*N* = 8/group).∗*p* < 0.05; ∗∗*p* < 0.01 Student’s t test. (D) Zymography from non- KC patients (male vs. female) (*N* = 5/group) ∗*p* < 0.05; ∗∗*p* < 0.01 Student’s t test. (E) Zymography from KC patients (male vs. female) (*N* = 5/group) ∗*p* < 0.05; ∗∗*p* < 0.01 Student’s t test. (F) Immunoblotting of PLA2G2A of tear fluids collected non- KC patients (male vs. female) (*N* = 5/group),∗*p* < 0.05; ∗∗*p* < 0.01 Student’s t test. (G) Immunoblotting of PLA2G2A of tear fluids collected KC patients (male vs. female) (*N* = 5/group),∗*p* < 0.05; ∗∗*p* < 0.01. Student’s t test. Data are represented as mean ± SEM. All raw gel blots normalized using SYPRO total protein stain (See Figures S3A–S3F).

Recent studies have found that plasma estrogen levels were significantly higher in patients with corneal ectasia (including KC and post-refractive surgery ectasia) compared to a control group.^15^^,^^30^^,^^31^ However, hormone levels in patient tear fluids have not been studied in the context of corneal ectasia. In this study, we collected and analyzed tear fluids from thirty-three patients with KC and compared to twenty-seven healthy controls of similar age, sex, race, and ethnicity (Table 1). We measured the tear estrogen levels using enzyme-linked immunosorbent assay (ELISA). KC patients were grouped on the basis of sex. We observed that female KC patients had significantly higher estrogen levels (*p* < 0.01) compared to non-KC patients (both male and female) (Figure 1A), and overall, KC patients had significantly higher tear levels of estrogen (*p* < 0.01) compared to non-KC controls (Figure S1A). The tear fluid estrogen level was normalized to total proteins. Because estrogen has been reported to regulate PLA2G2A activation,^32^ we evaluated tear fluid PLA2G2A levels and found that PLA2G2A was significantly higher in KC patients compared to non-KC patients (*p* < 0.01) (Figure 1B). When we analyzed the expression of MMP9 protein level in the tear fluids of KC and non-KC patients, MMP9 protein levels were significantly higher (*p* < 0.05) in the tears of KC patients (Figure 1C); these findings are aligned with the previously published reports.^17^^,^^18^ All tear protein normalization was performed using SYPRO Ruby total protein stain as previously reported.^33^

**Table 1:** Patient demographic details

	KC (*n* = 33)	Control (*n* = 27)	*p* value
Age, mean (SD), years	32.44 (8.6)	34.1 (4.2)	0.36
Male sex, no. (%)	21 (63.6)	13 (48.2)	0.22
White Race, no. (%)	16 (48.5)	7 (25.9)	0.11
Hispanic Ethnicity, no. (%)	7 (21.2)	3 (11.1)	0.49
KC Severity (Amsler-Krumeich)	Grade 4: 33% (*n* = 11)	–	–
	Grade 3: 39% (*n* = 13)		
	Grade 2: 21% (*n* = 7)		
	Grade 1: 6% (*n* = 2)

Since PLA2G2A and MMP9 were significantly upregulated in KC patients, we analyzed the sex-specific differences on the MMP activity using zymography and PLA2G2A protein levels. In non-KC patients there were no significant differences between males and females in NGAL-MMP9 complex gelatinase activity or pro-MMP9 gelatinase activity (Figure 1D). In contrast, NGAL-MMP9 complex gelatinase activity was significantly upregulated (*p* < 0.01) in KC females compared to KC males (Figure 1E). Furthermore, while we observed no sex-specific differences in PLA2G2A levels in non-KC patients, (Figure 1F), KC females exhibited significantly higher PLA2G2A levels (*p* < 0.01) than males (Figure 1G) Taken together, these findings indicate that KC females have significant upregulation of estrogen in tear fluid compared to non-KC patients (male or female), with notably higher levels of PLA2G2A and NGAL-MMP9 gelatinase activity compared to KC males.

### Sex-specific changes in tears cytokine profiles and the effect of KC and non-KC tears treatment on MMP2/9 and PLA2G2A axis in co-culture model

**Figure 2:**
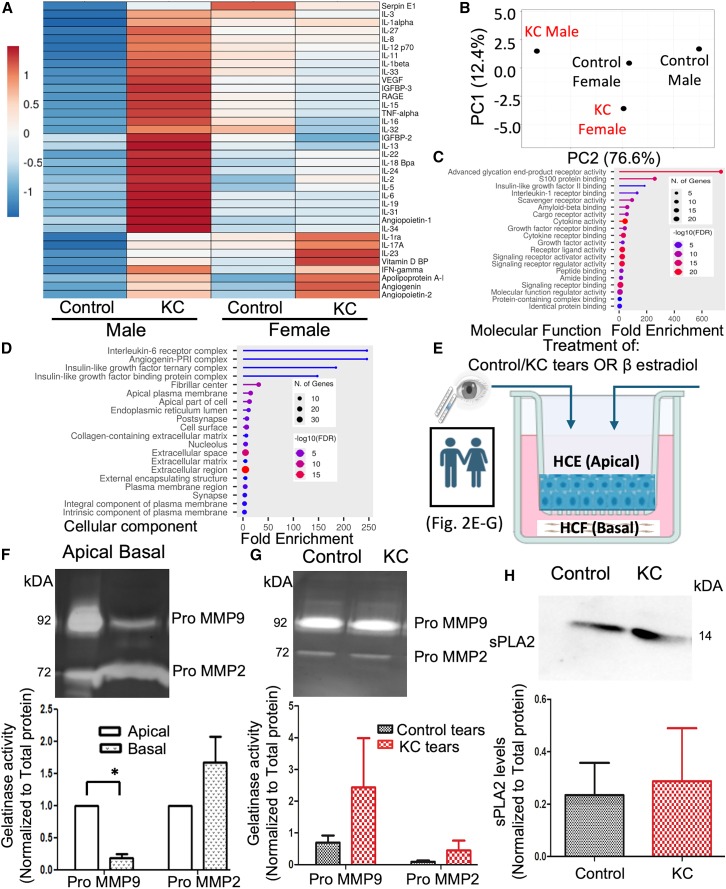
Sex-specific changes in tears cytokine profiles and the effect of KC and non-KC tears treatment on MMP2/9 and PLA2G2A axis in co-culture model (A) Cytokine array using proteome profiles of KC and non- KC patients (male vs. female) (*N* = 3/group). (B) Principal component analysis of KC and non- KC patients (male and female) (*N* = 3/group). (C and D) Pathway analysis cellular component and molecular function of KC and non- KC patients (male and female). (E) Pictorial representation of KC and non-KC patients tear fluids and β-estradiol treatment on the co-culture model apically. (F) Zymography of apical and basal conditioned medium collected from 7 days co-culture (*N* = 3–4 biological replicates) ∗*p* < 0.05; ∗∗*p* < 0.01 Student’s t test. (G and H) Zymography and immunoblotting of PLA2G2A of apical conditioned medium after treating with KC and non -KC tear fluids apically. (*N* = 3 each group), ∗*p* < 0.05; ∗∗*p* < 0.01 Student’s t test. Data are represented as mean ± SEM. All raw gel blots normalized using SYPRO total protein stain (See Figures S4A–S4D).

The sex specific differential regulation of MMP/PLA2G2A axis may play an important role in regulating corneal inflammation, as both MMPs and PLA2G2A are known to regulate inflammation.^34^ Since MMP9 and PLA2G2A protein levels were significantly upregulated in KC tears, we performed cytokine profiling of KC and non-KC tears and evaluated sex-based differences in cytokine expression. Males and females in both KC and non-KC patients exhibited distinguished cytokine profiles (Figure 2A) and the clinical details are presented in (Table 2). Overall male KC tear fluids had the highest number of upregulated cytokines and proteins compared to all others in the group (which include female KC patients and non KC patients both males and females).These cytokine and proteins include vascular endothelial growth factor A (VEGF-A), receptor for advanced glycation end product (RAGE), insulin growth factor binding protein 3 (IGFBP3), tumor necrosis factor-alpha (TNF-α), angiopoetin1, IL-2, -3, -5, -6, -8, -11, −13, −15, −19, −24, −27, −31, and −32, whereas the cytokines which were significantly upregulated in KC female tear fluids compared to others in the group includes IL-1-ra, IL-17a, and IL-23, vitamin D binding protein, interferon gamma, angiopoetin2, apolipoprotein, and angiogenin. Non-KC female tears also display a distinct cytokine profile compared to non-KC males, with upregulation of cytokines such as Serpin E1, IL-3, IL-32, TNF-α, and IL-12p70. These data indicates sex-based differences in the tear cytokine profile between both KC and non-KC patients; these findings are supported by previously published data indicating gender-based differences in the protein profiles of healthy tear donors^35^. Principal component analysis showed clustering on the basis of sex and disease (Figure 2B). Kyoto Encyclopedia of Genes and Genomes (KEGG) pathway enrichment analysis for the molecular function and cellular component using the expression pattern of cytokines revealed the IL-6 receptor complex, ECM remodeling, advanced glycation end product (AGE) receptor activity, IL-1 receptor binding activity and scavenger receptor binding activity be affected in KC pathogenesis (Figures 2C and 2D).

**Table 2:** Demographic details of patients for which cytokine array were performed (*N* = 3 males and 3 females per group)

	KC (*n* = 6)	Control (*n* = 6)	*p* value
Age, mean (SD), years	33.6 (6.3)	35.6 (2.4)	0.47
White Race, no. (%)	3 (50)	2 (33)	0.34
Hispanic Ethnicity, no. (%)	1 (16.7)	1 (16.7)	1
KC Severity (Amsler-Krumeich)	Grade 4: 50% (*n* = 3)	–	–
	Grade 3: 33% (*n* = 2)		
	Grade 2: 17% (*n* = 1)		
	Grade 1: 0% (*n* = 0)		

In a polarized co-culture model of immortalized human corneal epithelial cells and primary corneal fibroblasts (Figure 2E), apical and basal conditioned medium were analyzed for pro-MMP9 and MMP2 gelatinase activity. Pro MMP9 was secreted significantly more at the apical surface, while Pro-MMP2 was secreted significantly more at the basal surface (*p* < 0.05) (Figure 2F). These findings demonstrate the spatiotemporal patterns of MMP secretion in the co-culture model.

To understand the effects of tear fluids on the corneal epithelium secretion of MMP2, 9, and PLA2G2A, control and KC tear fluids were applied apically to the co-culture model and apical MMP9 and MMP2 activity were assessed. No significant changes in apical secretion of pro-MMP9 or MMP2 (*p* = 0.567) levels were observed in response to KC or non-KC tears (Figure 2G). KC and non-KC patients tear fluid treatment did not cause any significant changes in the apical pro-MMP9 or 2 gelatinase activity or PLA2G2A levels (*p* = 0.967) between control and KC tear fluid treatment (Figures 2G and 2H). The proteins levels were normalized using SYPRO Ruby total protein stain.

In summary, our data indicates sex-specific differences in cytokine and protein expression in both KC and non-KC patients, resulting in distinct clustering during principal component analysis. MMP9 and MMP2 secretions are regulated in a spatiotemporal manner in the co-culture model. Furthermore, treatment with KC and control tears on the corneal epithelium did not significantly affect pro-MMP9/MMP2 secretion or PLA2G2A levels, suggesting that the changes in tears may be a consequence of KC pathology, rather than a cause.

### Apical and basal β-estradiol treatment differentially regulates MMP2/9 secretion in co-culture model

The apical treatment of control and KC tear fluids to co-culture model did not significantly affect the MMP9/2 and PLA2G2A levels. To further elucidate the independent effect of β-estradiol, apical treatment of β-estradiol (mimicking tears) was given once daily for 3 days. Apical β-estradiol (10 pg/mL and 50 pg/mL) of treatment resulted in a significant increase in pro-MMP9 levels at the apical surface (*p* < 0.05) compared to untreated (Figure S1B). Gene expression of estrogen receptors (ERα, ERβ, and GPR 30) was assessed following β-estradiol (10 pg/mL and 50 pg/mL) treatment to elucidate the underlying mechanisms. Treatment with 10 pg/mL of β-estradiol significantly upregulated ERα expression (*p* < 0.05) with no significant changes observed in Erβ (Figure S1C).

**Figure 3:**
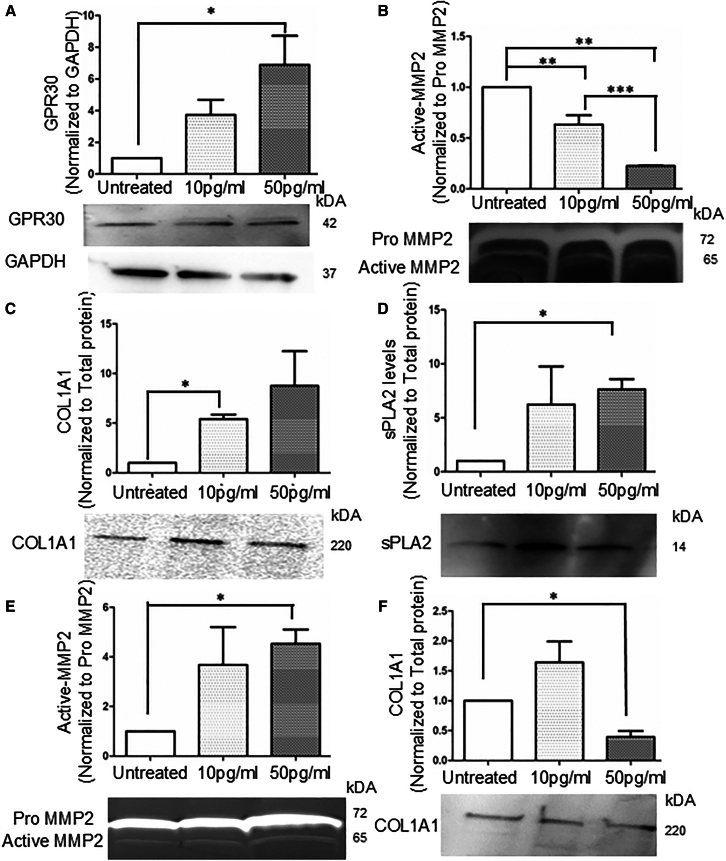
Apical and basal β-estradiol treatment differentially regulates MMP2/9 secretion in co-culture model (A) Immunoblotting of GPR30 in corneal epithelial cells after treating the co-culture apically with different concertation of β-estradiol (N = 3–4 biological replicates) ∗*p* < 0.05; ∗∗*p* < 0.01 one-way ANOVA. (B) Zymography of basal conditioned medium after Apical treatment of β-estradiol in co-culture model (N = 3–4 biological replicates), ∗*p* < 0.05; ∗ ∗*p* < 0.01 one-way ANOVA. (C) Immunoblotting of COL1A1 of the extracellular matrix after Apical treatment of β-Estradiol in co-culture model (N = 3–4 biological replicates), ∗*p* < 0.05; ∗∗*p* < 0.01 one-way ANOVA. (D) Immunoblotting of PLA2G2A of basal conditioned medium after Apical treatment of β-Estradiol in co-culture model (N = 3–4 biological replicates), ∗*p* < 0.05; ∗∗*p* < 0.01 one-way ANOVA. (E) Zymography of basal conditioned medium after basal treatment of β-estradiol in co-culture model (N = 3–4 biological replicates), ∗*p* < 0.05; ∗∗*p* < 0.01 one-way ANOVA. (F) Immunoblotting of COL1A1 of the extracellular matrix after basal treatment of β-estradiol in co-culture model (N = 3–4 biological replicates), ∗*p* < 0.05; ∗∗*p* < 0.01 one-way ANOVA. Data are represented as mean ± SEM. All raw gel blots normalized using SYPRO total protein stain (See Figures S5A–S5F).

Using the KC tear fluids cytokine data, we performed pathway analysis which showed that IL-6 receptor complex plays an important role in the pathogenesis (Figure 2C). Therefore, we analyzed the expression of IL-6 mRNA in corneal epithelial cells in response to β-estradiol treatment and found that IL-6 was significantly (*p* < 0.05) upregulated at 50 pg/mL of β-estradiol treatment compared to untreated (Figure S1D). Apart from the nuclear receptor, we also analyzed the expression of GPR30, a G protein coupled receptor of β-estradiol.^36^ 50 pg/mL of β-estradiol treatment significantly increased GPR30 expression (*p* < 0.05) compared to untreated (Figure 3A). These results suggest differential regulation of estrogen receptors in corneal epithelial cells in response to different concentrations β-estradiol.

Interestingly apical treatment of β-estradiol significantly downregulated the basal active MMP2 secretion in a concentration dependent manner (*p* < 0.01) compared to untreated (Figure 3B). As collagen1 is a substrate of MMP2, the downregulation of active MMP2 levels resulted in a significant accumulation of collagen 1 (COL1A1) in the ECM (*p* < 0.05) at 10 pg/mL of β-estradiol concentration compared to untreated (Figure 3C). Also, the apical treatment of β-estradiol (50 pg/mL) caused significant increase in expression of PLA2G2A (*p* < 0.05) compared to untreated (Figure 3D). As MMP2 deficient mice has been reported to have significantly higher levels of PLA2G2A,^34^ similarly apical β-estradiol caused significant downregulation of active MMP2 levels and upregulation of PLA2G2A levels indicating that β-estradiol regulates the MMP2-PLA2G2A axis.

Treatment of corneal epithelial cells with an PLA2G2A inhibitor for 24 h did not significantly alter IL-6 and TGF-β1 expression, suggesting that PLA2G2A inhibition has minimal impact on the regulation of these inflammatory cytokines in corneal epithelial cells (Figure S1E). Furthermore, basal β-estradiol treatment (mimicking stromal β-estradiol levels) caused significant upregulation of active basal MMP2 compared to untreated (*p* < 0.05) (Figure 3E). The increase in basal active MMP2 secretion resulted in a significant downregulation in the accumulation of COL1A1 in the ECM (*p* < 0.05) at 50 pg/mL of β-estradiol treatment compared to untreated (Figure 3F). The proteins levels were normalized using SYPRO Ruby total protein stain. Further to explore the site-specific differential role of β-estradiol in the coculture, we compared estrogen receptor expression (ERα, ERβ) between corneal epithelial cells and corneal fibroblasts. Our results (Figure S1F) show that corneal fibroblasts have significantly lower transcript levels of ERα and ERβ compared to corneal epithelial cells, which explains the differential regulation of active MMP2 in response to β-estradiol treatment.

Our data collectively indicates that β-estradiol treatment promotes the expression of its receptors in corneal epithelial cells. This activation leads to a significant increase in pro-MMP9 secretion at the apical surface, while basal active MMP2 secretion decreases. Consequently, there is a marked accumulation of COL1A1 in the ECM. However, when β-estradiol treatment is shifted from the apical to the basal surface, it results in a significant increase in active MMP2 secretion, causing degradation of COL1A1 in the ECM. This highlights the importance of the β-estradiol treatment site in the differential regulation of MMP2 activity and collagen accumulation in the ECM.

### β-estradiol regulates MMP2/9-PLA2G2A axis via p38 MAP kinase pathway in corneal epithelium

Estrogen receptors act as transcription factors and regulate gene expression in response to β-estradiol treatment. While MMP2/9 gene promoters do not have an estrogen responsive element,^22^ β-estradiol treatment still modulates MMP2/9 activity, indicating the involvement of a different cell signaling mechanism.

**Figure 4:**
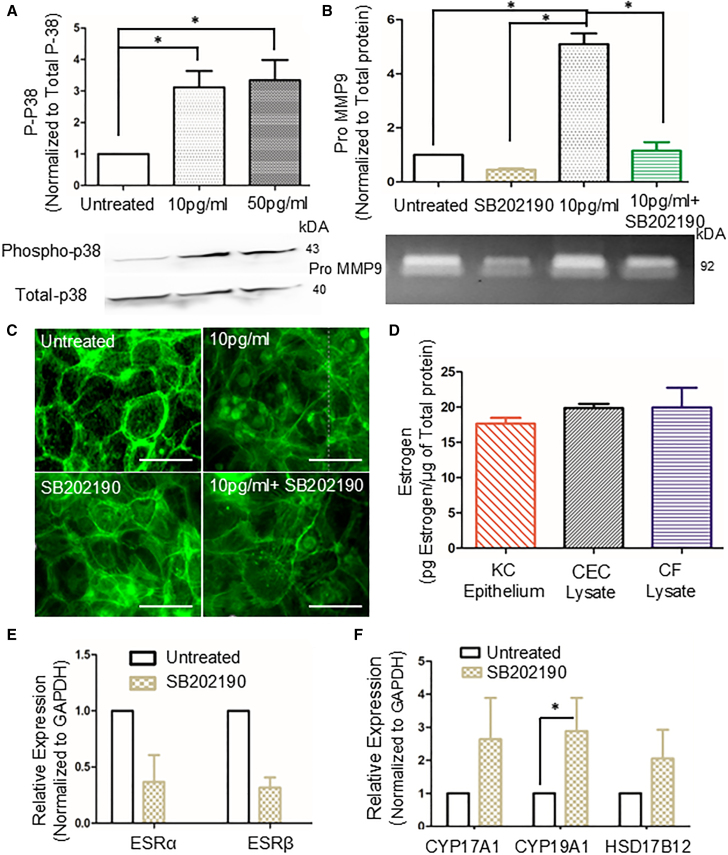
β-estradiol regulates MMP2/9 -PLA2G2A axis via p38 MAP kinase pathway in corneal epithelium (A) Immunoblotting of phospho p38 MAP kinase of corneal epithelial cells after apical treatment of β-estradiol in co-culture model (N = 3–4 biological replicates), ∗*p* < 0.05; ∗∗*p* < 0.01 one-way ANOVA. (B) Zymography of apical conditioned medium after apical treatment of estradiol alone and β-estradiol followed by SB202190 treatment in co-culture model (N = 3–4 biological replicates), ∗*p* < 0.05; ∗∗*p* < 0.01 one-way ANOVA. (C) Phalloidin staining of corneal epithelium after apical treatment of β-estradiol alone and β-Estradiol followed by SB202190 in co-culture model (*N* = 3 biological replicates) Scale bar: 20 μm. (D) Estrogen ELISA of KC corneal epithelium (collected during collagen crosslinking), corneal epithelial cells and corneal fibroblast (N = 3–4 biological replicates), ∗*p* < 0.05; ∗∗*p* < 0.01. (E and F) Real-time PCR of estrogen receptor and estrogen synthesis gene of corneal epithelium after apical treatment of SB202190 in co-culture model (N = 3–4 biological replicates), ∗*p* < 0.05; ∗∗*p* < 0.01 one-way ANOVA. Data are represented as mean ± SEM. Protein normalized using SYPRO. All raw gel blots normalized using SYPRO total protein stain (See Figures S6A and S6B).

Phospho-proteomic analysis of KC corneal epithelium has been reported to cause significant upregulate phospho-p38 protein levels.^23^ Therefore, we investigated the effect of apical β-estradiol on phospho-p38 levels in corneal epithelial cells. Apical treatment with β-estradiol (10 pg/mL and 50 pg/mL) in a co-culture model caused significant upregulation of phospho-p38 proteins in a concentration dependent way compared to untreated (*p* < 0.05) (Figure 4A). Since β-estradiol treatment activated phospho-p38 MAP kinase pathway and apical pro-MMP9 levels, we used SB202190—a p38 MAP kinase inhibitor— to evaluate its effects on β-estradiol mediated regulation of MMPs. Different concentrations of SB202190 (50 nM, 100 nM, and 1 μM) were treated to corneal epithelial cells for three days. After the treatment, Live/D-ead assay were performed (Figure S2A) showing that 50 nM of SB202190 were not toxic whereas 1 μM of SB202190 were highly toxic. Thus, we used 50 nM concentration for all our SB202190 treatment. Also, at a concentration of 50 nM,^37^ SB202190 significantly mitigated β-estradiol mediated secretion of pro-MMP9 (*p* < 0.05) (Figure 4B).

We analyzed the effect of β-estradiol on actin remodeling and distribution of the stress fibers using phalloidin staining because KC epithelium has been reported to have altered structures and actin remodeling.^24^ β-estradiol induced the formation of dorsal and ventral stress fibers in corneal epithelial cells compared to untreated cells, where the stress fibers were predominantly subcortical. Interestingly, SB202190 treatment alone induced dorsal and ventral stress fibers but when SB202190 were treated after β-estradiol treatment, it did not reverse the alteration in stress fibers induced by β-estradiol treatment (Figure 4C). So SB202190 was regulating the β-estradiol mediated activation of pro-MMP9 levels without affecting the actin stress fibers. To further investigate the role of cytoskeletal remodeling in MMP secretion, we treated corneal epithelial cells with 10 μM Y27632 (ROCK inhibitor) for 24 h. Our results (Figure S2B) showed no significant changes in pro-MMP9 or active MMP2 activity in Y27632-treated cells compared to untreated controls. Our data clearly demonstrates that corneal epithelial cells are responsive to β-Estradiol treatment.

We next performed an ELISA to determine if corneal epithelium from KC patients, corneal fibroblast from cadaveric donors and corneal epithelial cell line synthesize their own estrogen. Male KC corneal epithelium (collected during collagen crosslinking), along with corneal epithelial and fibroblast cells, secreted approximately 15–20 pg/μg of total protein (Figure 4D). Apical β-estradiol treatment led to a significant increase in pro MMP9 levels at the apical surface (Figure S2), whereas treatment with control and KC tear fluids did not induce any notable changes in the apical secretion of pro-MMP9 or MMP2 (Figure 2G). To determine whether endogenous estrogen production plays a more significant role than exogenous β-estradiol treatment, we utilized the aromatase inhibitor 1 (AI1) at 20 μM to block endogenous estrogen production, followed by supplementation with exogenous β-estradiol (10 pg/mL). Our data (Figure S2C) indicates that exogenous β-estradiol treatment in the presence of AI1 resulted in a significant downregulation of pro-MMP9 and active MMP2 levels compared to untreated controls. This suggests that both endogenous and exogenous β-estradiol contribute to increased pro-MMP9 and active MMP 2 activity, ultimately leading to corneal stromal collagen degradation.

SB202190 did not have any significant effect on estrogen receptors (ERα, β) (Figure 4E) or estrogen synthesizing genes (CYP17A1, HSD17B12) except CYP19A1, which is involved in aromatase enzyme synthesis and known to be regulated by p38 MAP kinase^38^ (Figure 4F).

Collectively, these findings suggest that β-estradiol modulates the phosphorylation of the p38 MAP kinase pathway, resulting in alterations to the orientation of stress fibers. While SB202190 inhibits β-estradiol-induced up-regulation of pro-MMP9, it does not impact the regulation of stress fibers by β-estradiol. Moreover, the corneal epithelium independently synthesizes its own estrogen, and SB202190 does not significantly affect the estrogen receptors pathway.

### SB202190 regulates β-estradiol-mediated collagen fiber degradation in corneal epithelium fibroblast 3D collagen hydrogel model

**Figure 5:**
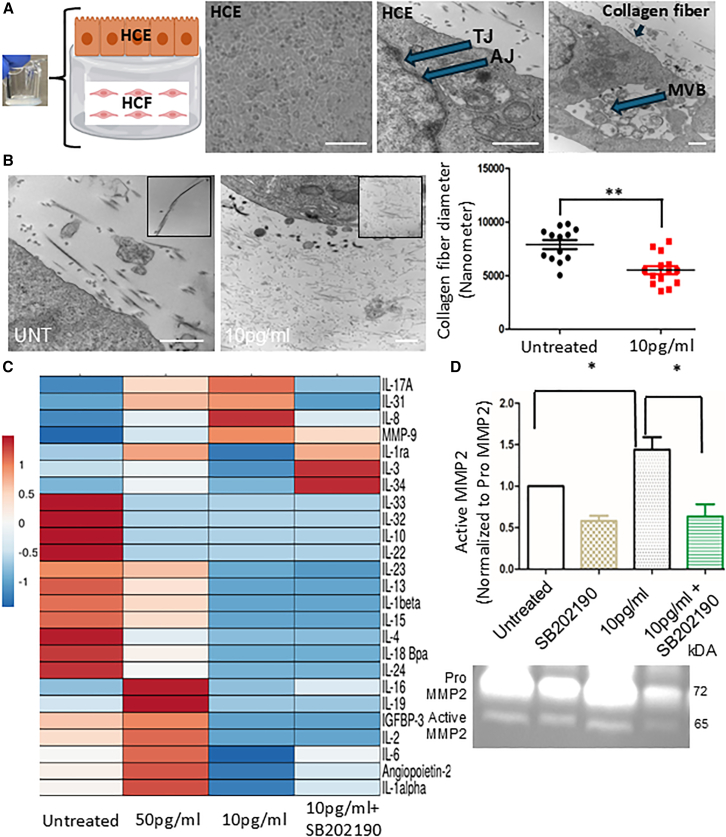
SB202190 regulates β-estradiol mediated collagen fiber degradation in corneal epithelium fibroblast 3D collagen hydrogel model (A) Pictorial representation TEM of 3D hydrogel with corneal epithelium, fibroblast, and collagen fibers. (B) TEM showing collagen fiber degradation and its quantification after treatment with β-estradiol (N = 3–4 biological replicates), ∗*p* < 0.05; ∗∗*p* < 0.1 Student’s t test Scale bar: 500 nm. (C) Cytokine array showing the effect of β-estradiol alone and β-estradiol followed by SB202190 treatment in 3D hydrogel model (*N* = 3). (D) Zymography of conditioned medium of 3D collagen hydrogel model after treatment of β-estradiol alone and β-estradiol followed by SB202190 treatment. (*N* = 3–4 biological replicates), ∗*p* < 0.05; ∗∗*p* < 0.01 one-way ANOVA All raw gel blots normalized using SYPRO total protein stain (See Figures S7A and S7B).

To study the effect of β-estradiol on corneal stromal collagen degradation, we developed a 3D collagen hydrogel model of the human cornea by encapsulating human corneal fibroblasts within a collagen hydrogel matrix and allowing human corneal epithelial cells to grow on top (Figure 5A). The collagen hydrogel had a Young’s modulus of 6 kPa, simulating that of the human cornea.^39^

Transmission electron microscopy (TEM) characterization revealed the formation of tight junctions (TJ), adherens junctions (AJ) between corneal epithelial cells, as well as the presence of multivesicular bodies (MVB) and collagen fibers (Figure 5A). We treated the hydrogel daily once for 3 days with β-estradiol and then performed TEM. We found that β-estradiol induced the breakdown of collagen fibers (Figure 5B), with the degradation occurring toward the fibroblast side. Quantification using Diameter ImageJ analysis revealed that β-estradiol induced a significant (*p* < 0.01) breakdown of collagen fibers.

Further we treated the hydrogel with different β-estradiol concentrations (10 pg/mL and 50 pg/mL) once daily for 3 days and then performed cytokine analysis of the collected conditioned medium. We found a dose dependent cytokine profile. Treatment with 50 pg/mL of β-estradiol resulted in the highest upregulation of cytokines, including interleukin-2, -6, -16, −19, −17A, −23, −31, -1α, IGFBP3, and angiopoietin-2. In comparison, treatment with 10 pg/mL of β-estradiol led to higher levels of MMP9, IL-8, IL-17, and IL-31, indicating a dose-dependent response of the hydrogel model to β-estradiol (Figure 5C). Interestingly, SB202190 was able to mitigate the β-estradiol (10 pg/mL) mediated upregulation of the cytokines and proteins (MMP9, IL-8, -31, and −17A). Most cytokines were upregulated at 50 pg/mL. However, MMP9 was upregulated at 10 pg/mL. Both MMP9 and MMP2 are regulated by β-estradiol in a spatiotemporal manner. To assess if SB202190 could inhibit this effect, we administered SB202190 followed by β-estradiol. This treatment aimed to determine whether SB202190 could block the β-estradiol-induced upregulation of MMP2. Our data showed SB202190 treatment reduced β-estradiol-induced active MMP2 activity by 1.5-fold in 3D collagen hydrogel model (Figure 5D), thus partially inhibiting the collagen fiber degradation.

In conclusion, our data suggests that the 3D collagen hydrogel model is a useful tool for studying hormone-mediated collagen fiber degradation. Using this model, estradiol treatment regulated cytokine secretion in a concentration dependent manner. Furthermore, the use of SB202190 can mitigate β-estradiol-induced regulation of MMP2/9 and prevent collagen fiber degradation.

## Discussion

In this study we found that estrogen levels are elevated in KC eyes, and that β-estradiol regulates MMP activity and ECM modeling in corneal co-cultures and corneal 3D collagen hydrogel models; this clinical and laboratory data suggests that β-estradiol may influence KC pathogenesis. It is well-established that the corneal epithelium is hormone-responsive, and our findings align with previous reports indicating that estrogen receptors, particularly ERα, are significantly upregulated in the KC epithelium.^40^ Notably, we observed that female KC patients exhibit significantly higher estrogen levels, NGAL-pro-MMP9 gelatinase activity and PLA2G2A levels than their male counterparts, with a corresponding increase in MMP9 protein levels. Sex-specific differences in MMP levels have been well documented in various systemic diseases, such as in cerebrospinal fluid of Alzheimer’s patients,^41^ plasma level of cognitive decline patients,^42^ serum levels in epilepsy,^43^ and in various other cardiovascular diseases.^44^^,^^45^^,^^46^

KC is a localized corneal ecstatic disease; however, it has been associated with various systemic conditions, including Down syndrome, atopy, osteogenesis imperfecta, Marfan syndrome, and mitral valve prolapse.^47^ A recent study compared tears and plasma, demonstrating that while many proteins and microRNAs overlapped between these biofluids, tears exhibited a distinct protein and microRNA profile.^48^ Given this, our study focused on tears rather than systemic biofluids to compare estradiol levels between KC and non-KC patients.

Previous studies have measured estrone, estriol, and 17β-estradiol levels in the saliva of KC patients and healthy controls,^9^ but not in tear fluid. They reported a significant downregulation of estrone in KC patients’ saliva, while estriol and 17β-estradiol levels showed no significant differences (*N* = 64). Additionally, no sex-specific differences were observed in estriol and 17β-estradiol levels between KC patients and controls. Similarly, another study in a cohort of 28 KC patients—a sample size comparable to ours—found no statistically significant association between corneal collagen crosslinking and plasma levels of estrone, estriol, and 17β-estradiol,^31^ suggesting that crosslinking does not influence systemic estrogen levels.

In our study, we observed a statistically significant increase in estradiol levels in the tear fluids of females with KC compared to female controls. This finding suggests that corneal changes may be more prominently reflected in tear fluids, given their anatomical relevance, whereas systemic biofluids may not optimally capture these changes. However, due to our relatively small sample size, the generalizability of these findings remains limited. Nonetheless, as noted earlier, previous studies have been conducted with similar sample sizes.^49^^,^^50^^,^^51^ Additionally, natural fluctuations in estrogen levels throughout the menstrual cycle (e.g., follicular, ovulatory, and luteal phases) and the use of contraceptives may influence hormone concentrations. Further research is needed to determine whether serum hormone levels affect hormone concentrations in other biofluids, such as saliva and tears. Little is known about tear fluids estrogen levels in KC eyes and the underlying mechanism of how estrogen may affect the cornea and the progression of corneal ectasia. Using tear fluids from KC and non-KC patients (male vs. female), and *in vitro* models (co-culture and 3D collagen hydrogel) of corneal epithelium and primary fibroblast our data offer valuable mechanistic insights into β-estradiol-mediated stromal collagen fiber degradation. KC female tear fluids exhibit significantly higher β-estradiol levels and a distinct cytokine profile. Additionally, the location of β-Estradiol treatment (apical vs. basal) influences MMP9/2 activity and activates the phospho-p38 MAP kinase pathway. Estrogen, a sex steroid hormone primarily produced by the ovaries, has also been found to be locally synthesized in various extragonadal tissues, including ocular tissues.^52^ It exerts its effects through three key receptors: ERα, ERβ, and GPR30. ERα and ERβ function as ligand-dependent transcription factors, collaborating with other transcription regulators to bind estrogen response elements within the promoter regions of numerous ECM genes—a collection documented in (https://estrogene.org/).

Estrogen functions not only as a transcription factor but also signals through GPR30, a G-protein-coupled receptor, activating downstream pathways such as MAP kinase and PI3K-AKT.^53^ These pathways modulate the activity of various proteases, including MMPs and phospholipases, as well as ECM components like collagen, proteoglycans, and fibronectin.^54^ Topical application of 17β-estradiol has been shown to significantly upregulate the mRNA expression of type I procollagen, tropoelastin, and fibrillin-1 by activating the TGF-beta receptor signaling pathway in an *in vivo* model of aged human skin. Additionally, it stimulates keratinocyte proliferation and enhances epidermal thickness, thereby improving connective tissue integrity and overall cutaneous function in aging skin^55^ In breast cancer, estrogen receptors (ERs) serve as critical biomarkers, with their expression in tumors influencing disease-free survival.^56^ In a mouse model of breast cancer, estradiol supplementation affects lysyl oxidase activity, which in turn regulates collagen fiber formation and orientation.^57^ Thus, both estrogen supplementation and local estrogen synthesis play a crucial role in ECM metabolism. By inhibiting the p38 MAP kinase pathway, β-estradiol-induced stromal collagen fiber degradation can be reduced, presenting a potential therapeutic approach. However further studies are necessary to understand the impact of other sex hormones, including progesterone, testosterone, and luteinizing hormone/follicle-stimulating hormone, on corneal stromal thinning.

MMPs have been extensively studied for their crucial role in the pathogenesis of KC,^16^^,^^17^^,^^58^^,^^59^^,^^60^ but few studies have explored their mechanism of actions and regulatory process. Increased levels of MMPs, particularly MMP1, MMP2, and MMP9, have been reported in KC corneal tissues and tear fluids; this suggests that there is dysregulated proteolysis and ECM remodeling.^16^ Our co-culture model of corneal epithelium and fibroblasts revealed that MMP9 was predominantly secreted at the apical surface, while MMP2 was higher on the basal side and that might explain why the KC tear fluids have higher level of MMP9 and not MMP2. Notably, treatment with KC and control tear fluids did not significantly affect pro-MMP9 activity or PLA2G2A levels, but β-estradiol treatment led to a significant upregulation of estrogen receptors (ERα and GPR30). Apical β-estradiol treatment activated pro-MMP9 activity at the apical surface while decreasing basal MMP2 activity, resulting in collagen I accumulation in the ECM. Conversely, when β-estradiol was applied to the basal surface, it activated basal MMP2 activity, leading to the inhibition of collagen accumulation. These findings indicate that β-estradiol modulates MMP activity via estrogen receptors, with distinct effects depending on the site of application.

Our study also highlights the role of GPR30 in mediating the effects of β-estradiol. GPR30 has been implicated in actin remodeling and the disruption of cell polarization,^61^ both of which have been previously reported in KC.^24^ We found that β-estradiol treatment regulated stress fiber formation in corneal epithelial cells, providing additional evidence of estrogen’s role in cellular polarization and ECM remodeling. The spatiotemporal regulation of MMP2 activity by β-estradiol underscores the complexity of its effects, with different outcomes depending on the location of receptor activation.

Cytokine profiling of KC tear fluids revealed sex-specific differences, with male KC patients exhibiting elevated levels of various cytokines, including IL-2, -5, -6, -19, and −31, TNF-α, and VEGF, compared to female KC patients and non-KC controls. Principal component analysis of these cytokine profiles revealed distinct clustering of male and female KC patients, while pathway analysis identified that these cytokines regulate key processes, such as IL-6 and IL-1 receptor complexes, ECM remodeling, and scavenger receptor activity. These findings suggest that cytokines play a critical role in the regulation of ECM remodeling and inflammation in KC. Moreover, the concentration-dependent effect of β-estradiol on cytokine levels observed in our hydrogel model provides insight into how estrogen may influence KC progression.

KC is a disease of complex etiology and there is no animal model to study the initiation or progression of the KC disease pathophysiology.^26^ The *in vitro* cell culture models derived from patient corneas are limited,^27^ as they cannot be used to study or quantify the collagen fiber degradation, which is one of the most common phenotypes observed in patients. Therefore, we developed a 6 kPa, 3D hydrogel model using corneal epithelial cells, collagen type 1, and primary corneal fibroblasts from cadaveric corneas to study factors driving KC and collagen fiber degradation. Our 3D collagen model of the corneal epithelium and fibroblasts is designed to closely mimic the anatomical structure of the cornea with corneal epithelial cells positioned on top of fibroblasts that are crosslinked within the underlying collagen hydrogel so that it is analogous to the corneal stroma. This model enables the quantification of collagen fiber degradation, emphasizing its significance in screening potential drugs for KC. However, this model lacks Bowman’s membrane, which could be incorporated to enhance its accuracy in future studies. The epithelium, ECM, and fibroblasts together replicate the native tissue environment, where autocrine, paracrine, and hormonal signals are selectively released by specific cell types, leading to spatiotemporal receptor-ligand interactions and expression. Unlike the human cornea, our ECM is not heterogeneous and consists solely of collagen type I. Nevertheless, the ECM secreted by different cell types contributes to its heterogeneity, effectively mimicking the corneal stromal environment. The hydrogel’s ECM not only provides chemical signaling but also offers essential mechanical support, as KC is a disease characterized by biomechanical instability.

Further, studies are needed to assess how hydrogel elasticity influences cell behavior and responses to KC-related factors.

Estrogen’s role in regulating MMP activity has been previously reported in other systems.^62^ Our study extends this knowledge by demonstrating that β-estradiol modulates MMP9 and MMP2 activity in corneal epithelial and fibroblast co-cultures via the p38-MAP kinase pathway. The spatial distribution of MMP9 and MMP2 activities in the polarized co-culture model highlights the complex regulation of MMPs in the cornea, with higher MMP9 activity at the apical side and higher MMP2 activity at the basal side. This nuanced regulation may be influenced by cellular interactions or local microenvironments, potentially having important implications for disease progression or targeted therapeutic strategies.

In addition to ECM remodeling, our study suggests that β-estradiol-mediated regulation of MMPs also influences inflammation. MMPs are key modulators of inflammation^63^ and many cytokines are substrates of MMP2.^64^ Our findings indicate that β-estradiol treatment differentially regulates cytokine levels, including IL-2, IL-5, IL-6, IL-19, IL-31, TNF-α, and VEGF. The concentration-dependent effects of β-estradiol on cytokine levels could help explain variations in disease progression at different stages of life that are associated with varying levels of estrogen. Future studies using cytokine inhibitors (IL-6R) which is a key therapeutic target for ocular inflammation are warranted but were beyond the scope of the current study.

Our findings are consistent with previous phospho-proteomic studies that have shown significant upregulation of MAP kinases in KC corneal epithelium.^23^ Other proteomic and bioinformatics studies have highlighted MMP2 and p38 MAP kinase as signature genes related to cell death in KC.^25^^,^^65^ Although β-estradiol’s regulation of p38 MAP kinase has been demonstrated in other systems, its role in corneal epithelium-fibroblast co-cultures and 3D hydrogel models has not been extensively studied. We found that β-estradiol spatiotemporally regulates MMP2 and MMP9 activity via the p38 MAP kinase pathway, and that SB202190, a p38 MAP kinase inhibitor, can reverse the estrogen-mediated regulation of these MMPs. Notably, SB202190 also restored the regulation of MMP2 activity in the 3D hydrogel model, suggesting that p38 MAP kinase inhibitors could be a promising therapeutic strategy for mitigating estrogen-induced collagen degradation in KC. However, future research is needed to explore how β-estradiol regulates other MAP kinase pathways.

Our multidimensional study provides a greater understanding of the role that estrogen may play in KC and how β-estradiol modulates corneal MMP activity, ECM remodeling, and inflammation. We also identified sex-specific differences in MMP activity, PLA2G2A levels, and cytokine profiles between male and female KC and non-KC patients. These findings add to our understanding of KC pathophysiology and may explain why KC first typically presents after puberty and why KC may progress with hormone replacement therapy in older patients who would be otherwise at low risk of progression. Further studies are needed to evaluate the efficacy and safety of p38 MAP kinase inhibitors in various models of KC.

### Limitations of the study

One major limitation of our analysis of KC tear fluids is the relatively small sample size, which may limit its generalizability. Serum estrogen levels fluctuate throughout the menstrual cycle and with contraceptive use. However, these factors were not accounted for when collecting tear fluid samples, potentially affecting estradiol levels, which is a limitation of this study. Whether serum estrogen levels influence tear estrogen concentrations remains uncertain and requires further investigation. Additionally, the effect of estradiol on hydrogel stiffness was not analyzed and needs further investigation. The potential of p38 MAP kinase inhibitors as a therapeutic option for estrogen-mediated corneal stromal thinning needs further validation. The 3D collagen hydrogel model used in this study lacks the Bowman’s membrane and is composed solely of type I collagen. Further research is warranted to incorporate the Bowman’s membrane and additional extracellular matrix components to better mimic the native corneal architecture.

## Resource availability

### Lead contact

Further information and requests for resources and reagents should be directed to and will be fulfilled by lead contact Joseph. B. Ciolino (joseph_ciolino@meei.harvard.edu).

### Materials availability

This study did not generate new unique reagents.

### Data and code availability

• Original western blot images have been provided in the supplementary figures.
• Microscopy data reported in this paper will be shared by the lead contact upon request.
• This study did not generate/analyze any datasets/code.
• Any additional information required to reanalyze the data reported in this paper is available from the lead contact upon request.

## Acknowledgments

This work was supported by National Institute of Health/National Eye Institute (NIH/NEI) grants [R01EY005665-37 (J.B.C.), K12EY016335-19 (L.N.K.)], and NIH Core Grant EY001792 and an unrestricted departmental grant from Research to Prevent Blindness (H.N.S), as well as the Schepens Core grant (P30EY003790), and generously supported by 10.13039/100011643Blavatnik Family Foundation (J.B.C.).

## Author contributions

Conceptualization: A.C. and J.B.C.; methodology and investigation: A.C., L.N.K., N.B., A.E.R., V.Y., N.V., H.S., and J.B.C.; visualization: A.C., L.N.K., N.B., A.E.R., V.Y., N.V., H.N.S., and J.B.C; formal analysis: A.C., L.N.K., N.B., A.E.R., V.Y., N.V., H.N.S., and J.B.C.; writing – original draft: A.C.; writing – review and editing: L.N.K., N.B., A.E.R., V.Y., N.V., H.N.S., and J.B.C.; project administration and supervision: J.B.C.; funding acquisition: J.B.C.

## Declaration of interests

Mass Eye and Ear is in the process of preparing a provisional US patent application: Drug Treatment for corneal inflammation and stroma thinning.

## STAR★Methods

### Key resources table

**Table:** 

REAGENT or RESOURCE	SOURCE	IDENTIFIER
**Antibodies**		

Anti Phospholipase A2	Abcam	ab23705, AB_2283834
MMP9	Cell Signaling Technology	13667, AB_2798289
Phospho-p38 MAPK (Thr180/Tyr182) (D3F9)	Cell Signaling Technology	4511, AB_2139682
p38 MAPK (D13E1)	Cell Signaling Technology	8690, AB_10999090
COL1A1 (E8F4L) XP	Cell Signaling Technology	72026, AB_2904565
GAPDH	Cell Signaling Technology	51332, AB_2799390
GPR30	Thermo Fisher Scientific	AB_2546123
Anti-rabbit IgG, HRP-linked Antibody	Cell Signaling Technology	7074, AB_2099233
Anti-mouse IgG, HRP-linked Antibody	Cell Signaling Technology	7076, AB_330924

**Biological samples**		

Tears from Keratoconus and non-Keratoconus patients	Patients at Mass Eye and Ear	NA
Cadaveric cornea	National Disease Research Interchange (NDRI; Philadelphia, PA, USA)	NA
Keratoconus corneal epithelium	Patients at Mass Eye and Ear (During collagen crosslinking)	NA

**Chemicals, peptides, and recombinant proteins**		

Defined Keratinocyte Serum free medium	Gibco	10744019
Bovine Pituitary extracts	Gibco	13028-014
EGF, Human Recombinant	Gibco	10450-013
Eagle’s Minimum Essential Medium (EMEM)	ATCC	30-2003
Fetal Bovine Serum (Heat Inactivated)	Gibco	10082147
Phalloidin	Cytoskeleton, INC	PHDG1
Trypsin-EDTA	Thermo Fisher Scientific	25300054
β Estradiol- Water soluble	Millipore Sigma	E4389
Penicillin Streptomycin Gluta MAX	Thermo Fisher Scientific	A5873601
SB202190	Millipore Sigma	559388
Phosphate Buffered Saline	Gibco	70011044
Y-27632	Millipore Sigma	SCM075
sPLA2 inhibitor	Cayman chemicals	17277
Aromatase inhibitor I	Millipore Sigma	18-254
Paraformaldehyde	Polysciences	18814-10
SYPRO™ Ruby Protein Blot Stain	Thermo Fisher Scientific	S11791
4–20%Mini-PROTEAN TGX Precast Protein Gels	Bio-sRad	4561094
Direct-Zol™ RNA micropep,RNA isolation kit	Zymo Research	R2060
iScript™ cDNA Synthesis Kit	Bio-Rad	1708891
iTaq Universal SYBR™ Green Supermix	Bio-Rad	1725121
RIPA buffer	Thermo Fisher Scientific	89901
Dimethyl Sulfoxide (DMSO)	Avantor	97063-136
Zymogram Renaturing Buffer	Thermo Fisher Scientific	LC2670
Zymogram Developing Buffer	Thermo Fisher Scientific	LC2671
Tween®-20	Millipore Sigma	P-9416
Triton-X	Millipore Sigma	T9284

**Critical commercial assays**		

Proteome Profiler Human XL Cytokine Array Kit	R&D Systems	ARY022B
Human Estrogen ELISA Kit	Abcam	ab285239
Novex 10% Zymogram Plus (Gelatin) gels	Thermo Fisher Scientific	ZY00100BOX
Photo Col	Advanced Biomatrix	5201

**Deposited data**		

Uncropped western Blots and zymography gels	N/A	N/A

**Experimental models: Cell lines**		

Araki-Sasaki immortalized corneal epithelial cell line	HCE-TJ 71	N/A
Corneal fibroblasts	Fibroblast derived from the cadaveric cornea in the laboratory	N/A

**Oligonucleotides**		

CYP19A1- Fwd	CACATCCTCAATACCAGGTCC	NA
CYP19A1- Rev	CAGAGATCCAGACTCGCATG	NA
CYP17A1- Fwd	ACCAGAATGTGGGTTTCAGC	NA
CYP17A1- Rev	CCTTGTCCACAGCAAACTCA	NA
HSD17B12-Fwd	CCTGTCCCACTCTTGACCAT	NA
HSD17B12-Rev	AAAGTTGGCTTCCGGATTTT	NA
ESR1-Fwd	AATCTGCAGGGAGAGGAGTTTGT	NA
ESR1-Rev	ACTCGGTGGATATGGTCCTTCTC	NA
ESR2-Fwd	AGGGTACATACTGGAATTGAG	NA
ESR2-Rev	AAATCTTTGACATGCTCCTG	NA
IL-6-Fwd	GCAGAAAAAGGCAAAGAATC	NA
IL-6-Rev	CTACATTTGCCGAAGAGC	NA
TGF-Beta1-Fwd	TGTACCAGAAATACAGCAAC	NA
TGF-Beta1-Rev	CAAAAGATAACCACTCTGGC	NA
GAPDH-Fwd	AATCCATCACCATCTTCCA	NA
GAPDH-Rev	TGGACTCCACGACGTACTCA	NA

**Software and algorithms**		

GraphPad Prism software	Dotmatics	https://www.graphpad.com/features
ImageJ (Fiji)	NIH	https://imagej.net/software/fiji/
Image studio	LI-COR	
Microsoft Excel	Microsoft	https://www.microsoft.com/en-us/p/excel/cfq7ttc0k7dx?activetab=pivot%3aoverviewtab
CFX Manager Software #1845000	Bio-Rad	https://www.bio-rad.com/en-us/sku/1845000-cfx-managersoftware?ID=1845000

**Other**		

Amicon® Ultra Centrifugal Filter, 3 kDa MWCO	Millipore	UFC500308

### Experimental models and subject details

#### Human subject enrollment

Enrollment was based on predetermined inclusion and exclusion criteria. Specifically, adults (18 years or older), healthy or diagnosed with KC, as well as those undergoing corneal crosslinking or keratoplasty were included. 33 KC patients and 27 healthy controls between the ages of 25–45 were recruited. Amsler-Krumeich staging was used to classify KC severity based on available structural parameters relevant to this molecular study, with Grade 1 indicating mild corneal thinning and astigmatism, Grade 2 showing increased thinning and steepening, Grade 3 characterized by pronounced ectasia and scarring, and Grade 4 representing severe keratoconus with extreme thinning and potential corneal opacification. The demographic details were presented as Tables 1 and 2.

#### Tear sampling

At the beginning of the study, subjects were briefly instructed on tear fluid sampling using Schirmer strips (SS). Once informed consent was obtained, an eye care professional (ophthalmologist or optometrist) collected tear fluid samples using an SS while wearing sterile, nitrile powder-free exam gloves. For each patient, new gloves were used to insert the tip of an SS (TearFlo, HUB Pharmaceuticals, USA) between the lid and the globe of the inferior temporal portion of the subject’s eye and away from the cornea. After 5 min, and with a new set of gloves, the SS were removed and placed in an individual, sterile, dry 4 × 6 inch x 2 mil plastic Ziplock bag (Cytiva, Whatman, USA). All samples were then transported on ice and stored at −80°C. All sampling was done without anesthesia or other eye drops. Subjects were allowed to open or close their eyes during SS sampling. Tear fluid samples were collected from both eyes for all methods, though only tears fluid sampled from the right eye were analyzed in our study. All samples were collected between 9 and 12 a.m. to control for intra-day variability.

#### Ethics

Human subjects research was performed in accordance with the Declaration of Helsinki; approval was granted by the Massachusetts General Brigham’s Institutional Review Board (IRB) (2020P001877). Samples were collected at Massachusetts Eye and Ear (Boston, MA). Subjects were educated on the study’s purposes, risks, and benefits. Informed consent was obtained before subject enrollment, and the privacy rights of human subjects were observed. Thus, we can confirm that all experiments were performed in accordance with relevant guidelines and regulations.

### Method details

#### Co-culture model and treatment of β-estradiol and tears

Araki-Sasaki corneal epithelial cell line (HCE-TJ 71)^66^ which is a immortalized cell line was cultured in Keratinocyte serum free media (SFM) (Gibco, Grand Island, NY, USA), supplemented with 5 ng/mL epidermal growth factor (EGF; Gibco), 0.05 mg/mL bovine pituitary extract (BPE; Gibco), and 1x penicillin-streptomycin solution (Gibco). Primary corneal fibroblasts were isolated from Human cadaveric corneas obtained from the National Disease Research Interchange (NDRI; Philadelphia, PA, USA). All research adhered to the tenets of the Declaration of Helsinki. Cells were cultured as previously described.^28^ After isolation, cells were plated in 6-well plates and grown to 75% confluency in Eagle’s minimum essential medium (EMEM) (American Type Culture Collection; Manassas, VA, USA) with 10% fetal bovine serum (FBS; Atlanta Biologicals; Flowery Branch, GA, USA) along with 1x antibiotic–antimycotic (Gibco). Corneal fibroblasts were seeded at the bottom of 24-well Transwell inserts (0.4 μm; Greiner Bio-One) and cultured for 3 days. Following this, HCE-TJ71 cells were seeded within the Transwell inserts. The co-culture was maintained for 7 days to allow the HCE-TJ71 cells to polarize and form tight and adherens junctions. Keratinocyte SFM medium was supplied apically, while EMEM medium was applied basally.

A β-estradiol water-soluble form (Millipore Sigma; USA) stock solution was prepared by diluting in water. Based on previous reports, control and KC stromal cells were treated with 10 and 50 pg/mL of β-estradiol to activate the estrogen receptor pathway, and the same concentrations were used in the present study.^67^ For apical treatment, β-Estradiol was further diluted in keratinocyte SFM, while for basal treatment, it was diluted in serum-free EMEM. Treatments were applied at concentrations of 10 pg/mL or 50 pg/mL,^68^ either apically or basally, once daily for 3 days. After treatment, apical and basal conditioned medium were collected and analyzed by zymography or immunoblotting. PLA2G2A inhibitor (Cayman Chemicals) stock solution was prepared using DMSO as manufacturer recommendation.10uM concentration of PLA2G2A inhibitor was treated to corneal epithelial cells for 24 h. Aromatase inhibitor 1 (Millipore Sigma) and Y27632 (Millipore Sigma) stock solution was prepared as manufacturer recommendation. 20uM concentration of aromatase inhibitor 1 and 10uM of Y27632 was treated to corneal epithelial cells for 24 h. SB202190 (Millipore Sigma) was diluted in DMSO by manufacturer recommendation and apical treatment was given for 24 h at 50nM after 10pg/ml of β-estradiol treatment as previously reported.^37^

Tear fluids were collected as described above and were concentrated using Amicon Ultra centrifugal filters (3K, Millipore Sigma) following the manufacturer’s instructions. Total proteins were measured using BCA according to manufacturer’s instructions and equal protein concentration of tear fluids were treated apically to corneal epithelial cells.

#### 3D hydrogel model of corneal epithelium and fibroblasts

A 6 kPa hydrogel was prepared using PhotoCol kit (Advanced Biomatrix following the manufacturer’s protocol, using the same concentrations of collagen, crosslinking agent, and incubation time. Briefly, corneal fibroblasts were mixed with the hydrogel components and allowed to crosslink. Once the hydrogel was formed, HCE-TJ71 cells were seeded on top. The hydrogel was cultured for 3 days before starting the treatment. β-estradiol was then applied at concentrations of 10 pg/mL or 50 pg/mL once daily for 3 days. After the treatment conditioned medium was collected and analyzed by zymography. SB202190 were treated as mentioned above.

#### Zymography

Conditioned medium (apical and basal side) was concentrated using Amicon Ultra centrifugal filters with an MWCO of 3KDa (Millipore Sigma). Following concentration, the samples were run using Novex zymogram plus gelatin protein gels (Thermo Fisher Scientific). The gels were developed using Novex zymogram renaturing buffer and developing buffer. The gel was stained using colloidal blue staining kit (Invitrogen) and destained according to manufacturer instructions. The gels were image using gel documentation system (G:Box Syngene). The activity of the enzymes were normalized using total SYPRO Ruby protein blot stain (Invitrogen) as previously reported.^33^ The quantification was done using Image Studio Lite Version 5.2 (Licor).

#### Isolation of extracellular matrix (ECM)

ECM was isolated from underneath corneal fibroblast cultures as previously described.^69^ In brief, corneal fibroblasts were detached non-enzymatically by incubating with 10 mM EDTA in 1X PBS at 37°C for up to 20 min. Once the fibroblasts were fully removed, the wells were treated with ECM isolation buffer (containing 1% SDS and 10% glycerol in Tris buffer, pH 6.8) along with a protease inhibitor cocktail (Millipore Sigma) for 30 min at 37°C. The ECM extracts were then precipitated using acetone and used for Western blot analysis.

#### Immunoblotting

Proteins from corneal epithelial pellets was isolated with Pierce RIPA buffer (Thermo Fisher Scientific) containing protease inhibitor cocktail.^70^ Protein quantification was performed using Pierce BCA Protein Assay Kit (Thermo Fisher Scientific) following manufacturer’s protocol. Samples were diluted in 4x Laemmli SDS sample reducing buffer (Thermo Fisher Scientific) and heated at 90^o^C for 10 min. Denatured protein samples were then resolved on 4–20% Tris-HCl gradient gels (Bio-Rad, California, USA) and subsequently transferred onto polyvinylidene difluoride (PVDF) membranes (Millipore Sigma). Prior to analyzing levels of specific proteins using anti bodies, total protein levels on the PVDF membranes post-transfer were evaluated using SYPRO Ruby protein blot stain (Thermo Fisher Scientific) using the manufacturer’s instructions. The membrane was then blocked for 1 h at room temperature in 5% (w/v) non-fat dry milk powder (NFDM) in TBST (20 mM Tris–HCl pH 7.5, 150 mM NaCl and 0.1% Tween 20). It was washed with TBST and incubated at 4°C overnight with the antibodies listed. After overnight incubation, the membrane was washed thrice for 5 min with TBST and further incubated in corresponding horse radish perioxidase (HRP)-conjugated Anti-rabbit and Anti-mouse secondary antibody). The secondary antibodies used were diluted to 10,000--fold in 5% NFDM (w/v) in TBST. After incubation, the membrane was again washed thrice for 5 min with TBST. HRP activity was detected using HRP substrate (Bio-Rad Cat No-1705061) in Gel Documentation system (G:Box Syngene).

#### RNA isolation and quantitative real-time PCR

Total RNA was isolated from corneal epithelial cells using Direct-Zol RNA microprep (Zymo Research, California, USA) following manufacturer’s instructions. The isolated RNA was then quantified and used for cDNA synthesis using iScript cDNA Synthesis Kit (BioRad) following the manufacturer’s instructions. Quantitative real-time PCR was subsequently performed using previously published primers for ER α, β, CYP17A1, CYP19A1, HSD18B12, IL-6, using SYBR Green (Bio-Rad) and CFX-Connect Real Time System cycler (Bio-Rad). GAPDH served as a loading control for gene expression analyses with gene expression calculated relative to 18S/GAPDH followed by normalization to untreated samples in each individual experiment. Data were analyzed using the Bio-Rad CFX Manager 3.1 software and Microsoft Excel.

#### Immunofluorescence

After treatment, corneal epithelial cells cultured on Transwell inserts were fixed with 4% paraformaldehyde and washed with phosphate-buffered saline (PBS). The actin fibers of the tissues were stained with 100 nM Phalloidin-488 (Cytoskeleton, Inc., Cat No-PHDG1) following the manufacturer’s instructions for permeabilization. For the Live/Dead assay, corneal epithelial cells were treated with different concentrations of SB202190 (50 nM, 100 nM, and 1 μM). After treatment, the medium was replaced, and the assay was conducted according to the manufacturer’s instructions. The stained samples were then imaged using a Leica DMi8 microscope.

#### Transmission electron microscopy

Corneal epithelial-fibroblast 3D collagen hydrogels cultured on Transwell inserts were fixed using a fixative containing 2.5% glutaraldehyde and 4% PFA in 0.1 M sodium cacodylate. The hydrogels were then embedded in epoxy resin and sectioned. Finally, the sections were imaged using a JEM-1220 transmission electron microscope (JEOL USA, Peabody, MA, USA).

#### Enzyme linked immunosorbent assay

Tear fluids from both KC and non-KC patients were concentrated using Amicon Ultra centrifugal filters (3K, Millipore Sigma) following the manufacturer’s instructions. Estrogen levels in the tear fluids, as well as in cell and tissue lysates, were quantified using ELISA (Abcam Human Estrogen ELISA Kit, ab285239), according to the provided protocol. Estrogen levels were then normalized to the total protein content, which was measured using the Pierce BCA Protein Assay Kit (Thermo Fisher Scientific).

#### Cytokine array using proteome profiler

Cytokine detection in the tear fluid of KC and non-KC patients, as well as in conditioned medium, was conducted using array-based technology Proteome Profiler Human XL Cytokine Array Kit (Cat no ARY022B, R and D Systems, Minneapolis, USA) following the manufacturer’s instructions. The total protein content of each sample was first measured using the Pierce BCA Protein Assay Kit (Thermo Fisher Scientific), as previously described. Membranes were then incubated with samples containing equivalent total protein, and the procedure was carried out according to the manufacturer’s protocol. Signal detection was performed via chemiluminescence using the gel documentation system (G:Box Syngene), and analysis was done using ImageJ 1.53k (National Institutes of Health, Bethesda, MD).

### Quantification and statistical analysis

The number of biological or technical replicates used in each experiment is specified in the figure legends. All experiments were conducted with at least three independent repeats. Western blot data is presented relative to either total protein (as measured by SYPRO blot stain) or to the housekeeping protein GAPDH. Active MMP2 activity is normalized to the Pro MMP2 activity. Protein arrays were normalized based on total protein content. For patient data, statistical analyses were performed using an unpaired t-test or Fisher’s exact test. Data are presented as mean ± SEM. Statistical significance was assessed using unpaired two-group t-tests or one-way analysis of variance (ANOVA) for comparisons across different treatment groups, performed with Microsoft Excel and GraphPad Prism software. A *p*-value of ≤0.05 was considered statistically significant.
