# Supplementary material for: Estrogen-mediated corneal collagen degradation in keratoconus
Source: iScience. 2025 Jun 25;28(8):113004. doi: 10.1016/j.isci.2025.113004 (PMC12274804; doi:10.1016/j.isci.2025.113004)
Supplement: Document S1. Figures S1–S7 [file mmc1.pdf]

iScience, Volume *28*

Supplemental information

Estrogen-mediated corneal collagen degradation in keratoconus

Amit Chatterjee, Levi N. Kanu, Nikolay Boychev, Amy E. Ross, Vincent Yeung, Nandini Venkateswaran, Hajirah N. Saeed, and Joseph.B. Ciolino

Supplemental Figures

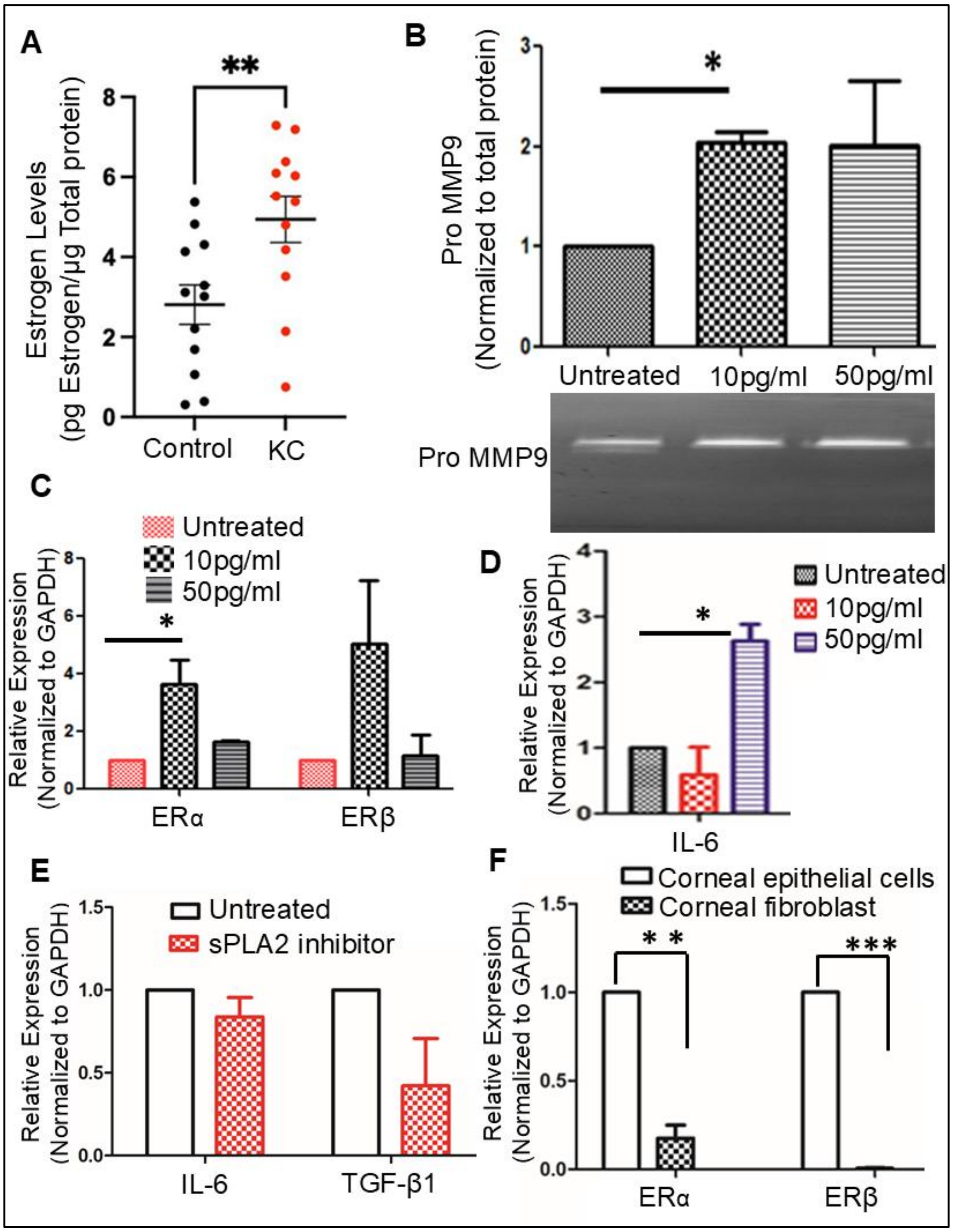

Supplemental figures S1 : Estrogen levels in tear fluid and the effects of estrogen treatment on estrogen receptor expression and MMP activity in corneal epithelial and fibroblast cells. **A**) ELISA of control and KC tears **B**) Zymography of apical conditioned medium after β estradiol treatment in corneal epithelial cells **C**) Real time PCR of Estrogen receptor ERα and β **D**) Real time PCR of IL6 after β estradiol treatment in corneal epithelial cells **E**) Real time PCR of IL6 and TGFβ-1 after sPLA2 inhibitor treatment in corneal epithelial cells **F**) Real time PCR of Estrogen receptor ERα and β between corneal epithelial cells and corneal fibroblast.

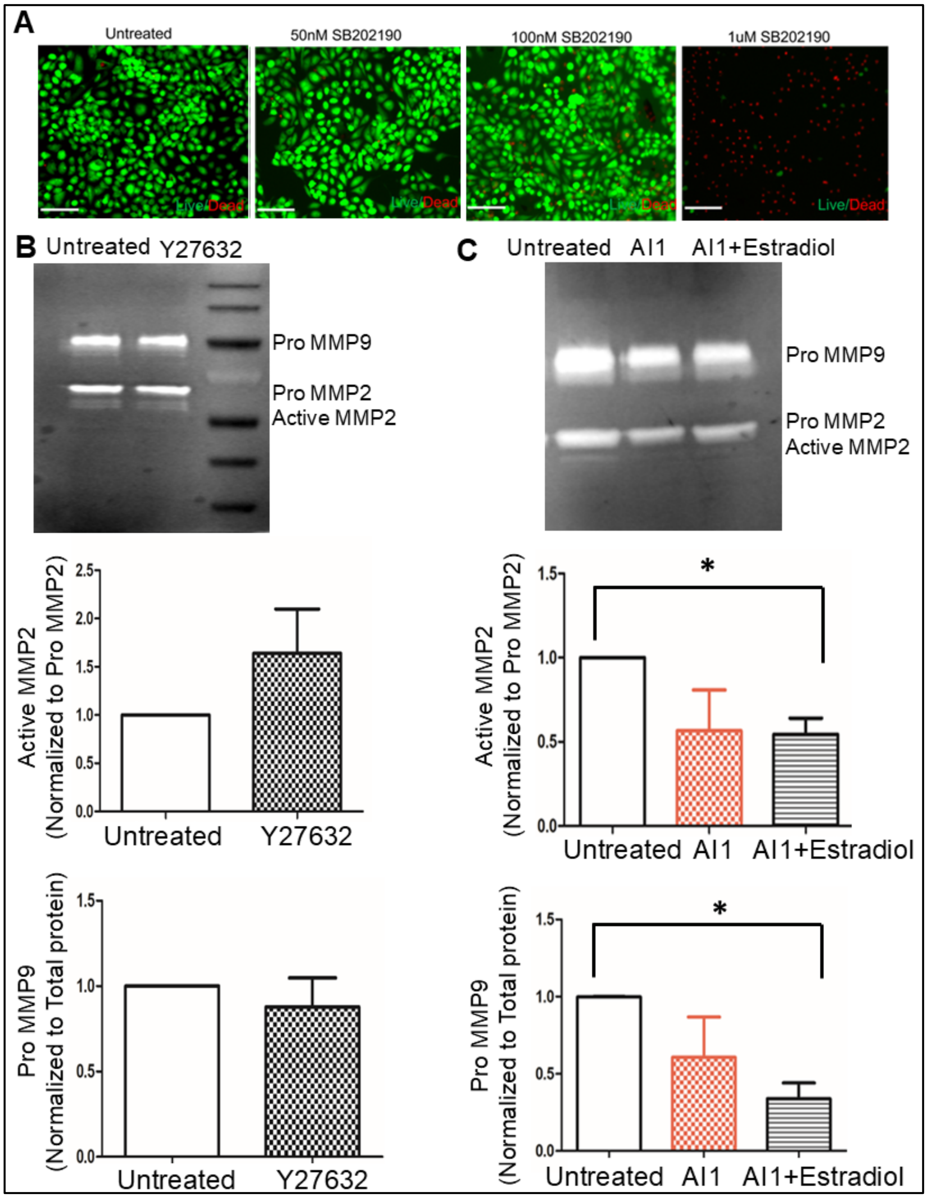

Supplemental figures S2: Use of inhibitors (ROCK inhibitor, Aromatase Inhibitor 1, and SB202190) to evaluate MMP activity. **A**) Live / Dead assay of corneal epithelial cells after 3 days of treatment with different concentration of SB202190. **B**) Zymography of conditioned medium after treatment with Y27632. **C**) Zymography of conditioned medium after treatment with aromatase inhibitor 1(AI1) and β estradiol supplemented with AI1 inhibitor.

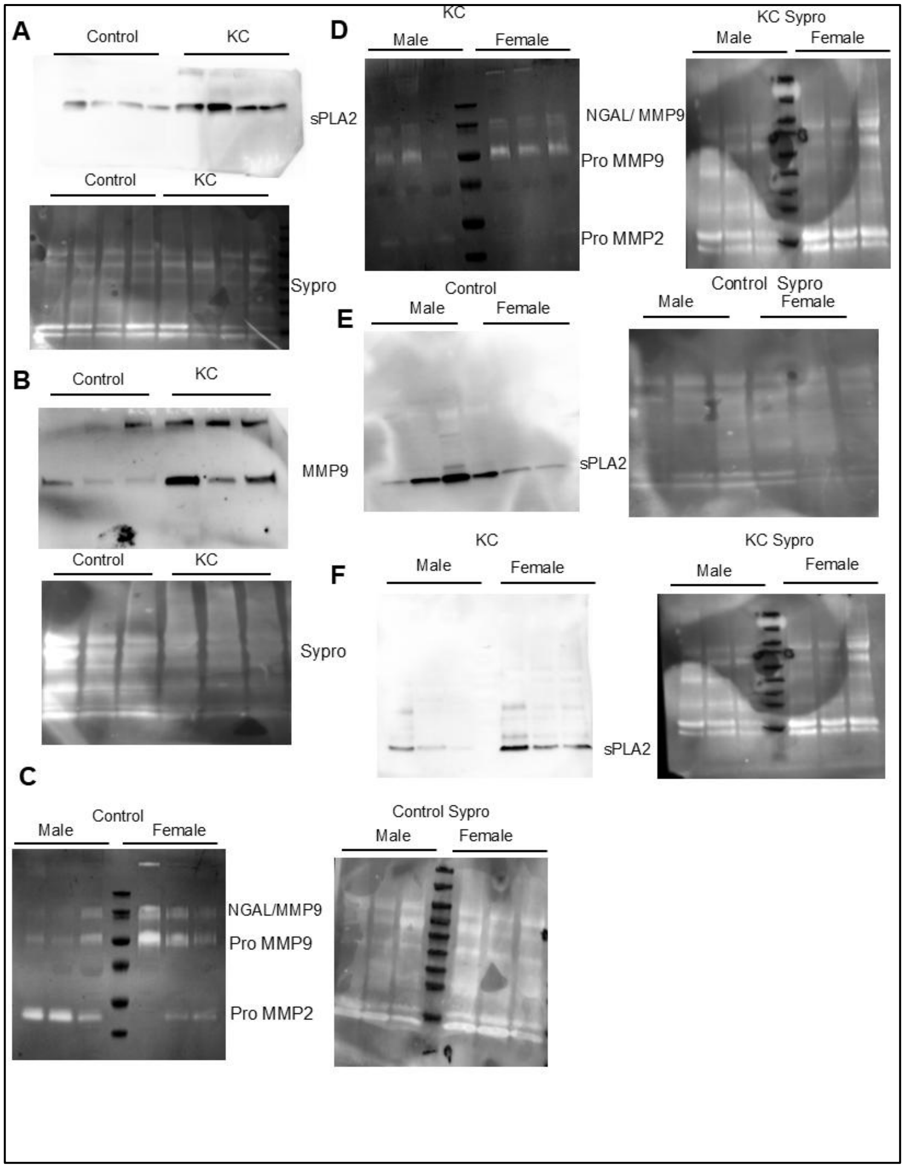

Supplementary Figure S3: Original Western blots and zymography gels of tear fluid samples. **A**) Raw blot of sPLA2 of control vs KC tears and sypro **B**) Raw blot of MMP9 of control vs KC tears and Sypro **C**) Zymography of control male vs female tears and sypro stain **D**) Zymography of KC male vs female tears and sypro stain **E**) Raw blot of sPLA2 of control Male vs female tears and sypro **F**) Raw blot of sPLA2 of KC Male vs female tears and sypro.

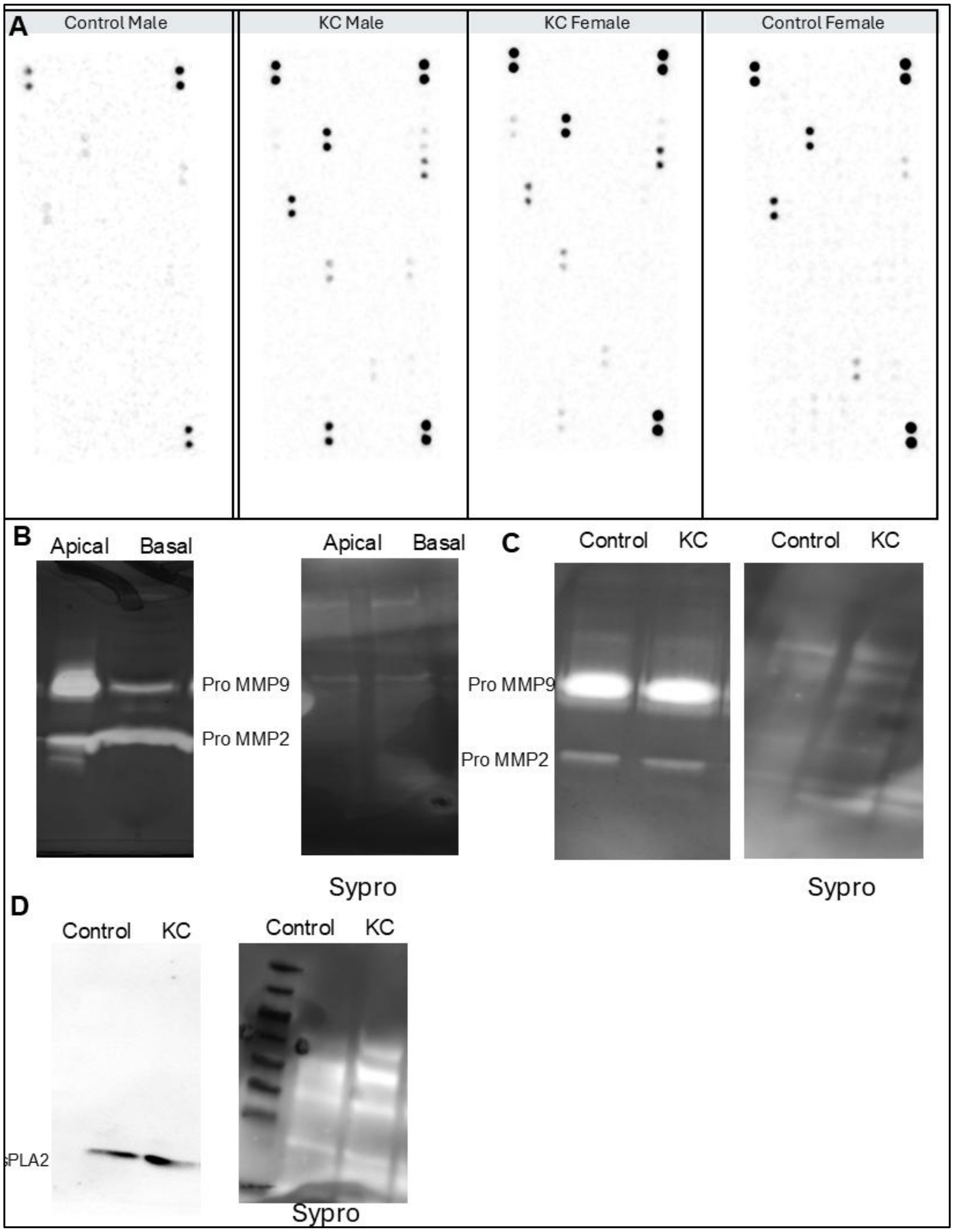

Supplementary Figure S4 : Unprocessed cytokine array and zymography gels of cell culture conditioned media. **A**) Representative Proteome profiler array of tears from control and KC patients **B**) Zymogram and sypro of apical and basal condition medium collected from co-culture **C**) Zymogram and sypro of conditioned medium collected after treatment with control and KC tears **D**) Raw blot of sPLA2 and sypro of control and KC tears treated apically to corneal epithelial cells.

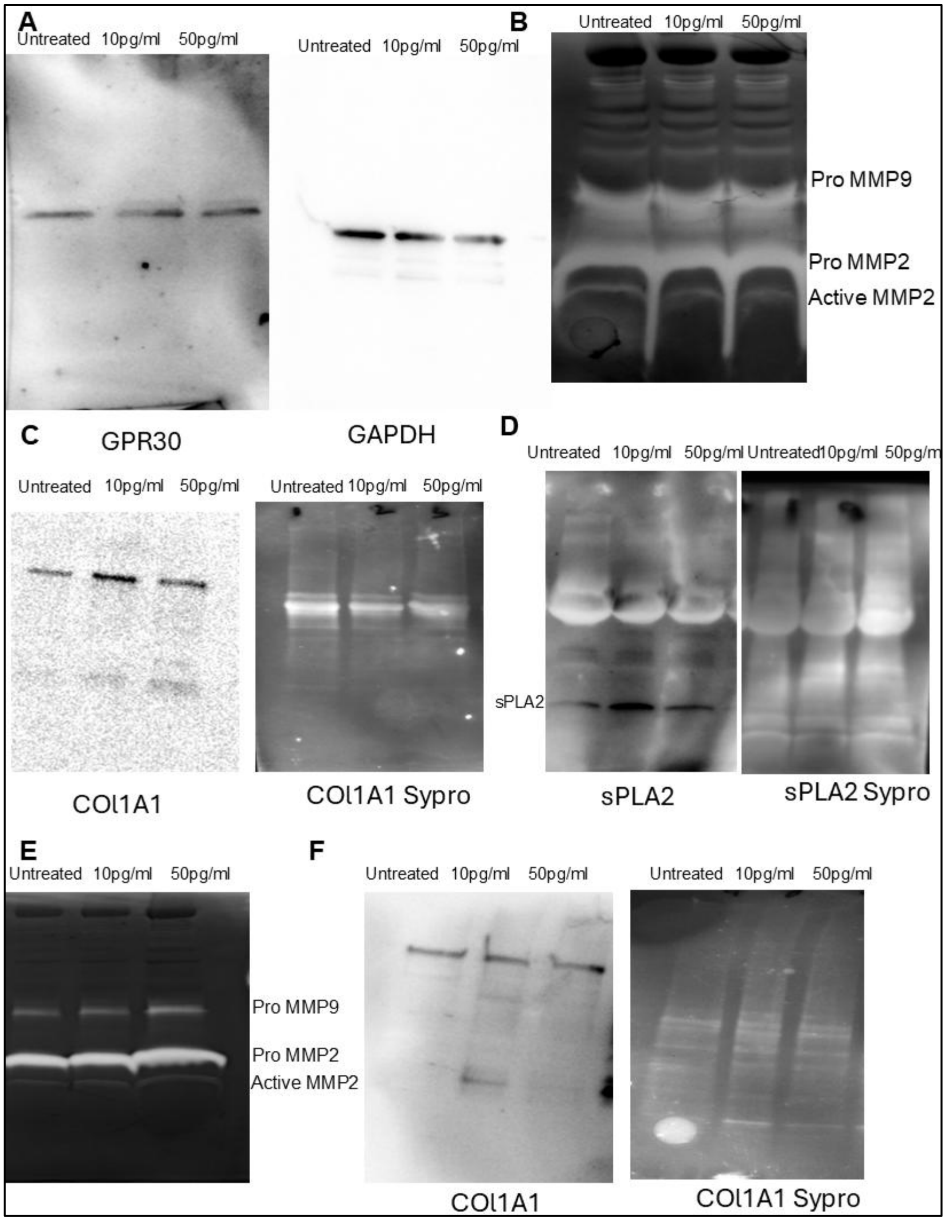

Supplementary Figure S5: Original Western blots and zymography gels of cell pellets and conditioned medium **A**) Raw blot of GPR30 and GAPDH in corneal epithelial cells after β estradiol treatment. **B**) Zymography of basal condition medium after apical β estradiol treatment **C**) Raw blot of COL1A1of ECM and sypro stain after β estradiol treatment **D**) Raw blot of sPLA2 of basal condition medium after β estradiol treatment **E**) Zymography of basal conditioned medium after basal treatment of β-Estradiol in coculture model **F**) Raw blot of COL1A1of ECM and sypro stain after β estradiol treatment.

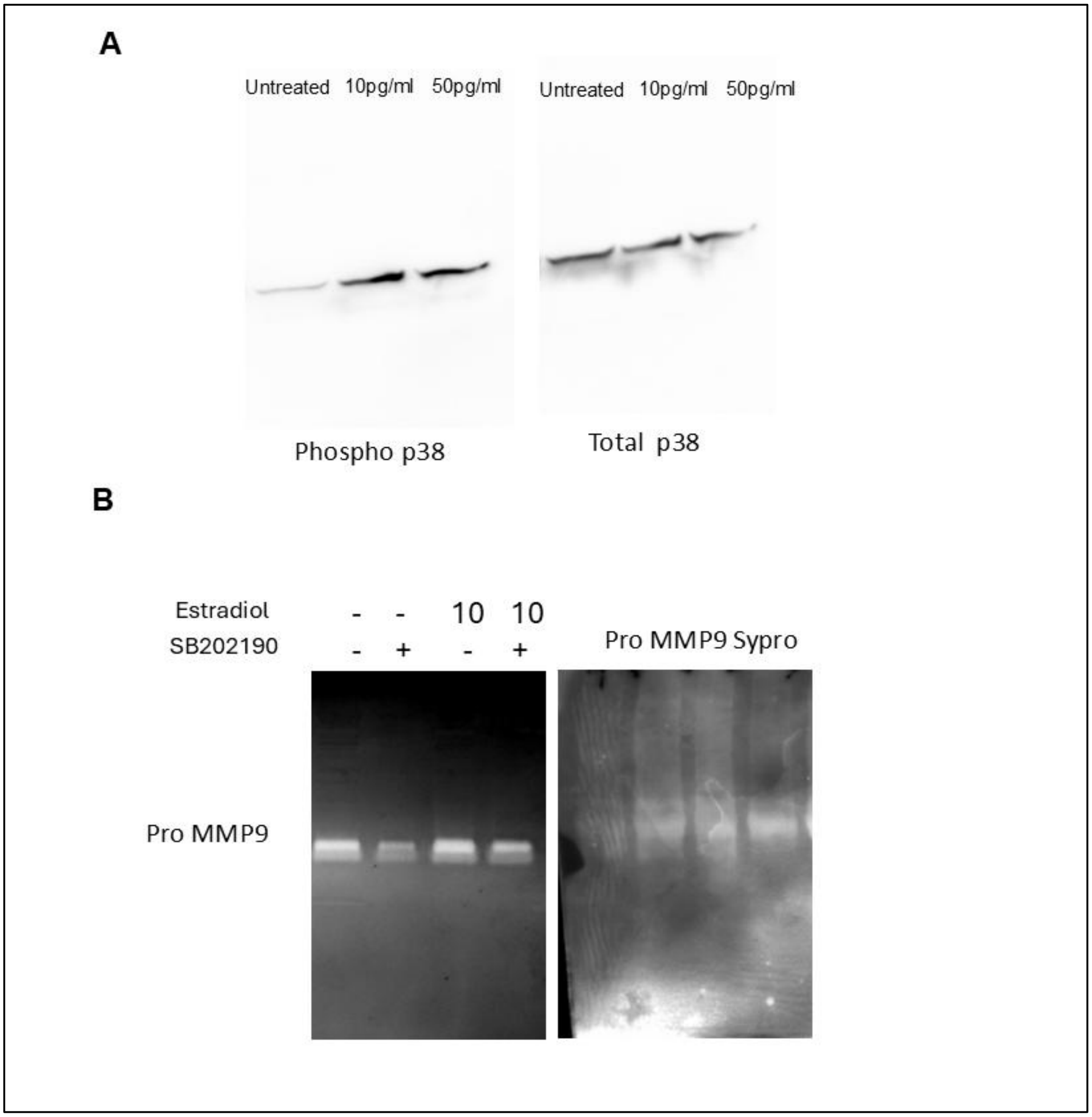

Supplementary Figure S6: Original Western blots and zymography gels of CEC pellets and Co-culture apical conditioned medium. **A**) Raw blot of Phospho p38 and Total p38 in corneal epithelial cells after β estradiol treatment. **B**) Zymography and sypro of apical conditioned medium after apical treatment of Estradiol alone and β-Estradiol followed by SB202190 treatment in coculture model.

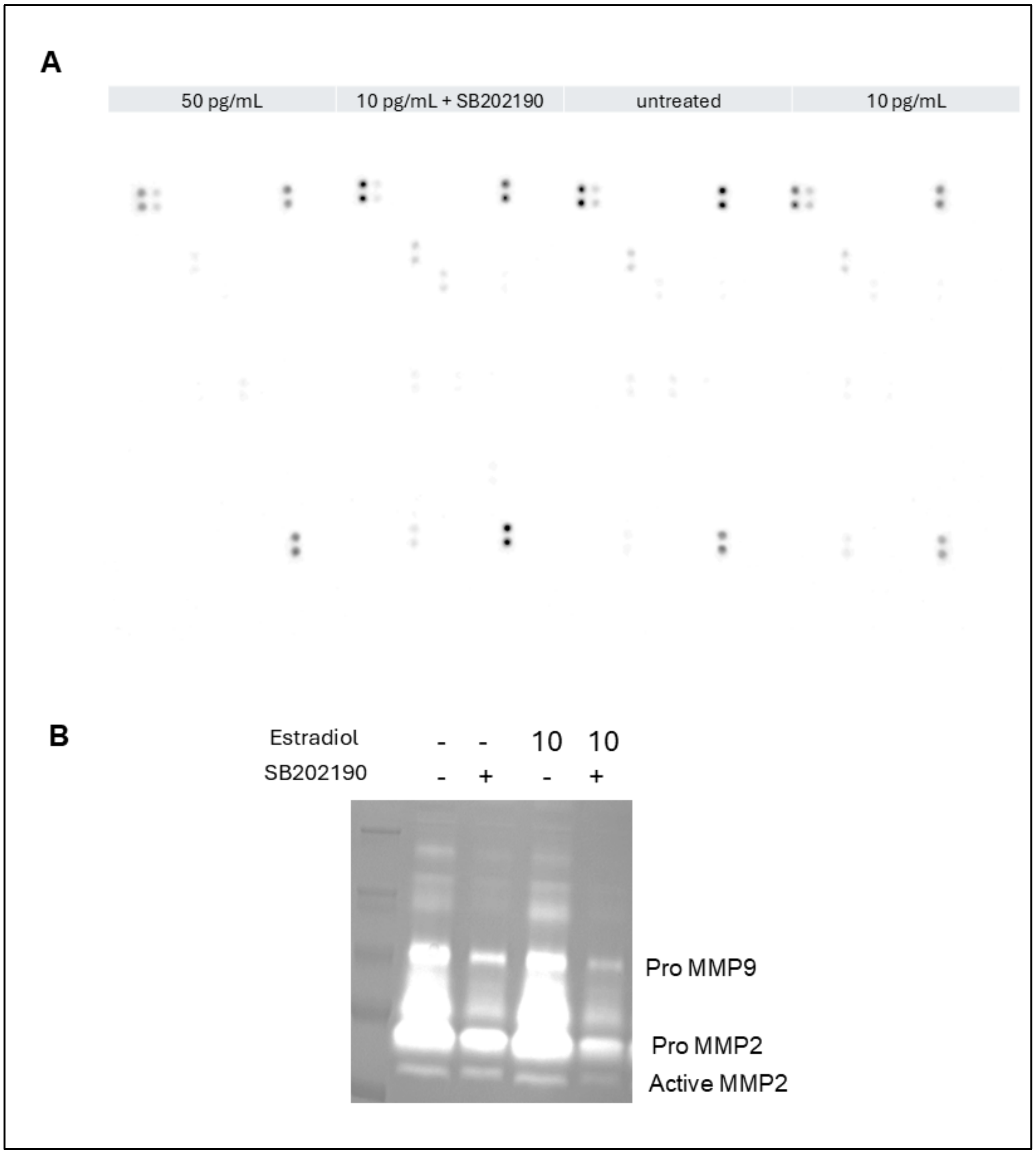

Supplementary Figure S7: Unprocessed cytokine array and zymography gels of conditioned medium collected from 3D corneal collagen hydrogel model. A) Representative Proteome profiler array of conditioned medium after treatment with β estradiol and SB202190 after β estradiol treatment B) Zymography of conditioned medium of 3D collagen hydrogel model after treatment of β-Estradiol alone and β-Estradiol followed by SB202190 treatment.
